# Colorectal Cancer Test Use Among Hispanic and Non-Hispanic U.S. Populations

**Published:** 2006-03-15

**Authors:** Lori A Pollack, Donald K Blackman, Katherine M Wilson, Laura C Seeff, Marion R Nadel

**Affiliations:** Division of Cancer Prevention and Control, National Center for Chronic Disease Prevention and Health Promotion, Centers for Disease Control and Prevention; Division of Cancer Prevention and Control, Epidemiology and Applied Research Branch, National Center for Chronic Disease Prevention and Health Promotion, Centers for Disease Control and Prevention, Atlanta; Division of Cancer Prevention and Control, Epidemiology and Applied Research Branch, National Center for Chronic Disease Prevention and Health Promotion, Centers for Disease Control and Prevention, Atlanta; Division of Cancer Prevention and Control, Epidemiology and Applied Research Branch, National Center for Chronic Disease Prevention and Health Promotion, Centers for Disease Control and Prevention, Atlanta; Division of Cancer Prevention and Control, Epidemiology and Applied Research Branch, National Center for Chronic Disease Prevention and Health Promotion, Centers for Disease Control and Prevention, Atlanta

## Abstract

**Introduction:**

Although colorectal cancer mortality rates in the general U.S. population declined slightly from 1992 to 2000, the rates for Hispanic men and women did not. Disparity in colorectal cancer screening among Hispanics may be an important factor in the unchanged mortality trends. This study examined rates of colorectal cancer test use among Hispanic and non-Hispanic adults in the United States.

**Methods:**

Using sampling weights and logistic regression, we analyzed colorectal cancer test use among 5680 Hispanic and 104,733 non-Hispanic adults aged 50 years and older who participated in the 2002 Behavioral Risk Factor Surveillance System. We estimated the percentages and adjusted odds ratios (ORs) of the respondents' reported test use by sociodemographic characteristics, health care access, and state or territory of residence.

**Results:**

Hispanic respondents aged 50 and older reported having had either a fecal occult blood test within the past year or a lower endoscopy (sigmoidoscopy or colonoscopy) within 10 years less frequently (41.9%) than non-Hispanic respondents (55.2%). Rates of test use were lower for respondents who reported less education, lower income, no health insurance, and no usual source of health care, regardless of Hispanic ethnicity. After adjusting for differences in education, income, insurance, and having a usual source of health care, Hispanic respondents remained less likely than non-Hispanic respondents to report colorectal cancer testing (OR for fecal occult blood test, 0.66; 95% confidence interval [CI], 0.56–0.81; OR for lower endoscopy, 0.87; 95% CI, 0.77–0.99). Greater disparity in screening rates between Hispanics and non-Hispanics was observed in Colorado, California, and Texas than in other states.

**Conclusion:**

A disparity exists between Hispanic and non-Hispanic U.S. adults in colorectal cancer test use. This disparity varies among the states, highlighting the diverse health care experience of Hispanic adults in the United States.

## Introduction

Colorectal cancer is a leading cause of cancer-related death in the United States (1). Although Hispanic men and women have lower incidence and mortality rates from colorectal cancer than non-Hispanics, colorectal cancer remains the second leading cause of cancer-related mortality among Hispanic men and third leading cause among Hispanic women (2). In 2003, 7000 new cases and 2300 deaths from colorectal cancer were expected among Hispanic men and women (2). Mortality rates among Hispanic individuals did not significantly decline from 1992 to 2000 despite reductions among non-Hispanic white and black men and women (3). Disparity in colorectal cancer screening among Hispanics may be an important factor in the unchanged mortality trends. Previous studies have shown that colorectal cancer screening rates are lower for Hispanic populations (4-8) and that Hispanic men and women are less likely to be diagnosed at an earlier stage than non-Hispanic whites (2).

Recommendations for colorectal cancer screening are that men and women of average risk begin regular screening at age 50 with one of the following: 1) an annual fecal occult blood test (FOBT), 2) a flexible sigmoidoscopy every 5 years, 3) a combination of FOBT annually with flexible sigmoidoscopy every 5 years, 4) a colonoscopy every 10 years, or 5) a double-contrast barium enema every 5 years (9-11). Supporting evidence for these screening tests is summarized in the recommendations from the United States Preventive Services Task Force (USPSTF) (12). Early detection and removal of precancerous polyps has been proven to prevent colorectal cancer incidence (13,14) and mortality (15). Several randomized controlled trials have demonstrated decreased colorectal cancer incidence (16) and mortality (17-19) through regular FOBT, and case-control studies have demonstrated the efficacy of sigmoidoscopy (20,21) and colonoscopy (13). Despite the effectiveness of screening, overall screening rates are low (22).

Identifying disparities in colorectal cancer testing among Hispanics could be useful in focusing interventions to reduce cancer mortality. The U.S. Hispanic population, representing 12.5% of the U.S. population, is heterogeneous, with each state having a unique composition of Hispanic subgroups and other demographic characteristics (23). Previous studies have examined colorectal cancer screening rates for Hispanic populations nationally (4-8) or within specific communities (24-28). However, comparisons of the results across communities are limited by differences in study design and questions (29). The Behavioral Risk Factor Surveillance System (BRFSS) is a national population-based survey of health behaviors designed with a standardized format to allow for state-based comparisons. A comparative analysis of colorectal cancer test use by Hispanic ethnicity across the United States and among states with large Hispanic populations has not been done previously.

We analyzed data from the 2002 BRFSS to estimate Hispanic-specific rates for receiving colorectal cancer tests. We determined national percentages and odds ratios (ORs) of Hispanic and non-Hispanic adults who reported having had colorectal cancer testing within recommended screening periods. We then examined state-specific test use rates among Hispanic adults in Puerto Rico and nine states that administered the survey in Spanish and had an adequate number of Hispanic respondents. Finally, to explore ethnic disparities within these states, we examined differences in the percentage of reported colorectal cancer test use between Hispanic and non-Hispanic respondents.

## Methods

The BRFSS is a national standardized, continuous random-digit–dial telephone survey begun in 1984 to monitor the behavioral risk factors associated with mortality and morbidity (30,31). The BRFSS is supported by the Centers for Disease Control and Prevention and conducted by all states, the District of Columbia, and three territories. The study population for the BRFSS includes civilian, noninstitutionalized adults aged 18 years and older with telephones. For the 2002 BRFSS, the median response rate, calculated according to procedures recommended by the Council of American Survey Research Organizations (CASRO) (32), was 58.3% (ranging from 42.2% in New Jersey to 82.6% in Minnesota). Poststratification weights by age, race, and sex of the state population account for the coverage of nonrespondents or ineligible households.

Respondents aged 50 years and older were asked questions about the use of tests that screen for colorectal cancer. Background knowledge of whether the respondent "ever heard of" the specific test was not assessed before asking about test use. The use of an FOBT was determined by asking, "A blood stool test is a test that may use a special kit at home to determine whether the stool contains blood. Have you ever had this test using a home kit?" If the respondent answered yes, the question was followed by, "How long has it been since you had your last blood test using a home kit?" Similarly, for sigmoidoscopy and colonoscopy, respondents were asked, "Sigmoidoscopy and colonoscopy are exams in which a tube is inserted in the rectum to view the bowel for signs of cancer or other health problems. Have you ever had either of these exams?" and "How long has it been since you had your last sigmoidoscopy or colonoscopy?"

Both sigmoidoscopy and colonoscopy are procedures that involve using a flexible, fiber-optic instrument (endoscope) to view the rectum and colon for abnormalities. Colonoscopy examines the entire colon, whereas sigmoidoscopy focuses on the lower third of the colon. In this report, we refer to both procedures collectively as *lower endoscopy*. BRFSS questions do not differentiate between sigmoidoscopy and colonoscopy. The recommended screening interval is every 10 years for colonoscopy and every 5 years for sigmoidoscopy; we defined reported use of lower endoscopy within the past 10 years as "within the recommended interval." Although the FOBT and lower endoscopy are tests that are commonly recommended for colorectal cancer screening (11), the BRFSS does not distinguish between testing used for screening and testing used for diagnostic purposes; therefore, we use the terms colorectal cancer *testing* and *test use* rather than *screening*.

Hispanic individuals are defined by the U.S. Census Bureau as individuals who indicate their origin as Mexican, Puerto Rican, Cuban, Central or South American, or other Hispanic origin (23). For the BRFSS, Hispanic ethnicity was determined by the question, "Are you Hispanic or Latino?" followed by questions about race. Because Hispanic ethnicity and race were separate questions, individuals reporting Hispanic ethnicity could be of any race. The BRFSS survey did not ask respondents to identify their Hispanic subgroup. Additional BRFSS variables used for this study include sex, age, highest grade of school completed (education), annual household income (income), having any kind of health care coverage (insurance), and having a personal doctor or health care provider (usual source of health care). All responses were self-reported.

The total number of 2002 BRFSS respondents aged 50 years and older in all states, territories, and the District of Columbia was 111,036. Of these respondents, 5680 identified themselves as Hispanic. Respondents who answered "Don't know/Not sure" or "Refused" to ever having had an FOBT (1.6%) or lower endoscopy (2.1%) were excluded. In addition, 1.2% of respondents were excluded because they were unsure or refused to answer questions about Hispanic ethnicity, education, insurance status, and usual source of care.

Statistical estimates were produced using SAS 8.02 (SAS Institute Inc, Cary, NC) and SUDAAN 7.5.3 (Research Triangle Institute, Research Triangle Park, NC). All data were weighted by age, sex, and race to reflect the population of each state. Percentages and 95% confidence intervals (CIs) were age-adjusted using 5-year age groups to standardize to the 2000 U.S. census. Using logistic regression, we calculated crude ORs and CIs for receiving recommended colorectal cancer testing, then adjusted the ORs for age, sex, race, Hispanic ethnicity, education, insurance, income, and having a usual source of care. The respondents who chose not to give information on income were grouped into an "unknown" category to be included in the logistic regression.

The state-specific analysis was limited to states and territories where the survey was administered in both Spanish and English and where there were at least 100 Hispanic respondents aged 50 or older. These states and territories were Arizona, California, Colorado, Florida,  Massachusetts, New Jersey, New Mexico, New York, Puerto Rico, and Texas. Of the 5680 respondents who reported Hispanic ethnicity, 3808 (67%) lived in one of the included states. It was not possible to determine from national BRFSS survey data which language was used during the interview (33). Therefore, we could not separately analyze surveys completed in Spanish and surveys completed in English.

## Results

### Colorectal cancer test use by Hispanic ethnicity 

Among both Hispanic and non-Hispanic respondents, a higher percentage of respondents reported having had a lower endoscopy test than having had an FOBT ever and within the recommended screening intervals (Table 1). The estimated percentage of Hispanic respondents aged 50 and older who reported having had an FOBT within the past year was significantly lower (12.5%) than for non-Hispanic respondents (22.3%). This finding was also true for lower endoscopy within 10 years (36.2% for Hispanics compared with 45.9% for non-Hispanics) or either test within recommended intervals (41.9% for Hispanics compared with 55.2% for non-Hispanics).

### Sociodemographic characteristics and health care access 

A higher percentage of non-Hispanic men than women reported having received colorectal cancer testing, whereas there was no difference by sex observed for Hispanic respondents (Table 2). For non-Hispanics, FOBT or lower endoscopy test use increased by 10-year age groups then declined after age 80. For Hispanic respondents, reported use of FOBT increased with each 10-year age group until 80 years then declined, whereas use of lower endoscopy continued to increase in each age group without decline.

Both non-Hispanic and Hispanic respondents who reported more education and a higher household income reported higher percentages of having had either an FOBT within 1 year or lower endoscopy within 10 years. Of note, the difference in the percentages of non-Hispanic and Hispanic respondents who reported lower endoscopy within 10 years was smallest among respondents in the highest income category. Respondents without insurance or without a usual source of care had the lowest percentage of reported colorectal cancer test use, regardless of ethnicity. Because of the small number of Hispanic respondents who were uninsured, the percentage for FOBT use within 1 year among this group could not be reported. The small number of respondents would lead to an unstable estimate. Among non-Hispanic respondents who reported no insurance and no usual source of care, the percentage of use of either colorectal cancer test within recommended intervals was 34.7% (data not shown).

### Crude and adjusted ORs

Table 3 presents the adjusted ORs and CIs from a logistic regression analysis of having reported receiving an FOBT within the past year or a lower endoscopy within 10 years by ethnicity, education, insurance, and usual source of care. Crude ORs showed that respondents who reported less education, no health insurance, or no usual source of care were less likely to report receiving appropriate colorectal cancer testing for FOBT and lower endoscopy (data not shown). When adjusted to account for age, sex, race, education, income, insurance, and having a usual source of care, Hispanic respondents remained less likely than non-Hispanic respondents to report having had either test within recommended screening intervals.

### Variations in colorectal cancer test use among Hispanic respondents 

The percentage of Hispanic respondents aged 50 and older who reported an FOBT within the past year ranged from 8.5% in Texas to 24.1% in Massachusetts, and the percentage of Hispanic respondents who reported a lower endoscopy within the past 10 years ranged from 28.9% in Colorado to 47.1% in Massachusetts (Table 4). For receiving either test within the recommended period, Hispanic respondents in the Northeastern states (Massachusetts, New Jersey, and New York) had higher testing rates than Hispanic respondents in the Western states (Arizona, California, Colorado, and New Mexico). In Arizona, the higher rates of overall reported test use (47.1%) is likely due to higher reported use of the FOBT. Among respondents living in Puerto Rico, such a small number reported ever having had an FOBT that a reliable estimate could not be given.

The Figure compares the percentages of respondents who reported receiving either an FOBT or a lower endoscopy within recommended screening intervals by Hispanic ethnicity. Percentages of Hispanic men and women who received recommended testing were generally lower than those of non-Hispanic individuals in each state evaluated, except for Massachusetts, where rates were similar. In contrast, there is a notably larger disparity between Hispanic and non-Hispanic groups in Colorado, California, and Texas than in the other states.

**Figure 1:**
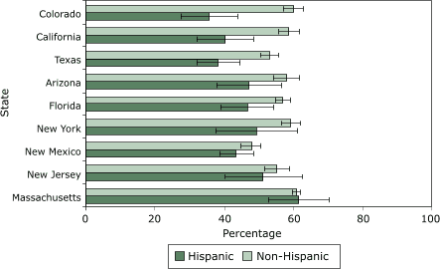
Age-adjusted percentage of Hispanic and non-Hispanic adults aged 50 years and older in selected states who reported receiving colorectal cancer screening (fecal occult blood test within past year, lower endoscopy within 10 years, or both) as recommended, Behavioral Risk Factor Surveillance System, 2002. Lines within bars represent 95% confidence intervals. Puerto Rico had too few non-Hispanic respondents to include in the comparison.

**Table:** 

## Discussion

The results of this study confirm that, regardless of ethnicity, colorectal cancer test use among men and women aged 50 years and older is low compared with other well-known cancer screening tests (4-6,34). We estimate that 42% of Hispanic respondents reported having had the recommended colorectal cancer testing compared with 55% of non-Hispanic respondents. This finding of lower screening rates for Hispanic men and women than for non-Hispanic men and women is consistent with other studies (4-8,24-26,34,35). Despite the relation of education, income, insurance, and a usual source of care to reported test use, adjusting for these factors does not fully account for the lower percentage of reported colorectal cancer test use among Hispanics. The adjusted analysis suggests that there are factors beyond health care access that prevent Hispanic men and women from receiving colorectal cancer tests.

This study also shows that Hispanic colorectal cancer test use differs among states. Colorado, California, and Texas had low percentages of Hispanic respondents reporting recommended test use and a greater disparity in colorectal cancer testing between Hispanic and non-Hispanic respondents than other states. In Massachusetts and New Jersey, test use among Hispanic men and women was higher than other states, and the disparity between Hispanic and non-Hispanic respondents was less. For states that had too few Hispanic BRFSS respondents for this analysis, colorectal test use and disparities are unknown. Often, Hispanic populations are analyzed as a single group, and these state-specific differences are not apparent. In addition to variation among states, there may be variation within each state as well. For example, Coughlin et al suggested that Hispanic women living in counties along the U.S.–Mexico border, regardless of their state of residence, were less likely to receive breast and cervical cancer screening than Hispanic women in nonborder counties (36).

A factor that may account for state differences is the unique composition and culture of Hispanic subgroups in each state. In general, the Western states have an overall higher Hispanic proportion of the population and, in the Western United States, Hispanic individuals are more likely to be of Mexican descent than they are in the Northeast, which has Puerto Rican, South American, and Central American populations (23). Hispanic subgroups could not be reflected in this analysis because BRFSS respondents who reported Hispanic ethnicity were not asked to report their subgroup. However, a recent study of the 2000 National Health Interview Survey (NHIS) analyzed cancer screening among Latino subgroups. Similar to our results, Sheinfeld Gorin and Heck found that FOBT use among Hispanic populations was low (14%) (37). In the subgroup study, a higher percentage of Mexican and Puerto Rican respondents (15%–22%) reported FOBT use than Cuban, Dominican, Central American, or South American respondents (9%–11%) (37). The same study showed endoscopy use within 5 years was highest among Cuban respondents (25%) and lowest among Dominicans, Central Americans, and South Americans (14%) (37). Our finding that screening rates are lower in the Western states, where the predominate Hispanic subgroup is Mexican, contrasts with the NHIS findings that Mexican respondents were more likely to have an endoscopy within the past 5 years.

Understanding the individual influences of ethnicity, socioeconomic factors, and health care access on colorectal cancer test use is challenging. Previous studies cite the lack of a usual source of health care as the most consistent reason that Hispanic individuals are not being screened for colorectal and other cancers (38-40) and also indicate that Hispanic men and women who receive other prevention services are more likely to be screened regularly (7,8,24,27). This study demonstrated that men and women without a usual source of health care are less likely to receive recommended colorectal cancer testing. However, studies that have attempted to separate socioeconomic factors and health care access from ethnicity in different populations have had differing results. Two separate studies in Washington State and the San Francisco Bay area reported that fewer Hispanic men and women reported receiving colorectal cancer tests than non-Hispanic individuals, but the differences disappeared when they adjusted for health care access and education (24,28). In contrast, a study in Texas reported that Hispanic women were less likely than non-Hispanic women to have ever received colorectal cancer screening by FOBT after adjusting for similar socioeconomic factors (41). Our findings, which represent a larger, national population, support the latter study that Hispanic men and women remain less likely to be screened despite education, income, and health care access.

The BRFSS survey did not ask whether Hispanic respondents were born outside of the United States and, if so, time since immigration to the United States. Therefore, we were not able to assess the role of acculturation and language of BRFSS respondents in this study. Analyses of other national health behaviors surveys have shown acculturation to be associated with reduced colorectal cancer rates (7). However, the effect of acculturation and language on screening for cervical and breast cancer has not always been shown to be as significant as access factors (8,42).

There were several limitations to this study. Actual compliance with screening guidelines may be lower than percentages reported in this article. In our study, to fully capture the use of colonoscopy, having a lower endoscopy (which refers to both sigmoidoscopy and colonoscopy) "within 10 years" is considered to be within recommended screening intervals. With this definition, individuals who received sigmoidoscopy outside the recommended 5-year screening interval but within 10 years were considered compliant with screening guidelines. In addition, there was no way to differentiate between tests performed for screening purposes and tests performed for diagnostic purposes. Also, the use of a double-contrast barium enema, an acceptable but less often recommended choice (43), was not available from the BRFSS survey. Comparisons of the rates in this report with other colorectal cancer screening studies must be made with caution because the time frame used for having received screening varies (29); some studies have examined whether respondents have ever been tested, while other studies have asked respondents about tests administered within the time frame of screening guidelines.

Telephone survey data are limited by several factors. The response rate to the 2002 BRFSS was 58%. Low response rates may bias results by selecting a unique population that differs from the general population in health care access, use, and beliefs. Despite this low response rate, BRFSS data have been shown to be valid and reliable when compared with other national surveys, and bias in demographic characteristics of respondents in BRFSS data was not associated with response rate (44). Additionally, the BRFSS excludes individuals who do not have household telephones and households that use a cellular phone exclusively. Low-income Hispanic households are less likely to own telephones than other low-income households (45) and may be underrepresented. Some Hispanic respondents may have experienced linguistic barriers to understanding the survey questions or may have answered the questions without having been familiar with the colorectal cancer tests. The U.S. Census Bureau estimates that more than one third of the more than 21 million people aged 18 and older who speak Spanish at home reported that they spoke English "not well" or "not at all" (46).

Responses were self-reported. The BRFSS questions did not ascertain whether the respondents had "ever heard of" the colorectal cancer test of interest. Despite previous studies, which have shown moderate and good concordance between self-reported colorectal cancer testing when validated with medical records (47,48), cognitive testing has shown that respondents have difficulty comprehending colorectal cancer questions because they often do not recognize or understand particular colorectal cancer tests (49). In this study, the responses were not validated, and respondents may not have been familiar with the test, may have felt a positive response was socially desirable, or both (29).

Colorectal cancer test use is estimated to be lower among Hispanic than non-Hispanic adults. Differences in test use cannot be fully explained by education, income, and health care access because, after adjusting for these factors, Hispanic men and women remain less likely to report having had colorectal cancer testing at recommended intervals. Certain Western states had a large disparity between the percentages of Hispanic and non-Hispanic test use, whereas Northeastern states had similar percentages of use. Regardless of the differing degrees of disparities, increasing awareness of and access to colorectal cancer screening among Hispanics is needed. The differences in colorectal cancer test use among states call attention to the diverse health care experience of Hispanic adults in the United States. Future studies that explore the reasons for differences in test use among Hispanic communities may highlight effective programs and practices that encourage increased screening.

## Figures and Tables

**Table 1:** Percentage^a^ of Respondents Aged 50 Years and Older (N = 110,413) Who Reported Receiving Colorectal Cancer Tests, Ever and Within Recommended Screening Periods, by Test Type, Behavioral Risk Factor Surveillance System, 2002

**Test Type**	**All Respondents, % (95% CI^b^)**	**Non-Hispanic Respondents, % (95% CI^b^) **	**Hispanic Respondents, % (95% CI^b^)**
FOBT^c^ ever	45.1 (44.6-45.6)	47.2 (46.7-47.7)	21.7 (19.3-24.1)
Lower endoscopy ever	49.2 (48.7-49.7)	50.1 (49.6-50.6)	39.9 (37.1-42.7)
FOBT or lower endoscopy ever	65.8 (65.3-66.3)	67.4 (66.9-67.9)	49.0 (46.2-51.8)
FOBT within last year	21.5 (21.1-21.9)	22.3 (21.9-22.7)	12.5 (10.4-14.6)
Lower endoscopy within 10 years	45.0 (44.5-45.5)	45.9 (45.4-46.4)	36.2 (33.4-39.0)
FOBT within last year or lower endoscopy within 10 years	54.0 (53.5-54.5)	55.2 (54.7-55.7)	41.9 (39.0-44.8)

a Age-adjusted to the 2000 U.S. census.

b CI indicates confidence interval.

c FOBT indicates fecal occult blood test.

**Table 2:** Percentage^a^ of Non-Hispanic and Hispanic Respondents Aged 50 Years and Older (N = 110,413) Who Reported Receiving Colorectal Cancer Tests Within Recommended Screening Intervals, Behavioral Risk Factor Surveillance System, 2002

**Characteristic**	**No. of Respondents**		**FOBT^b^Within 1 Year**		**Lower EndoscopyWithin 10 Years**	

	**Non-Hispanic**	**Hispanic**	**Non-Hispanic,% (95% CI^c^)**	**Hispanic,% (95% CI^c^)**	**Non-Hispanic,% (95% CI^c^)**	**Hispanic,% (95% CI^c^)**
**Total**	104,733	5680	22.3 (21.9-22.7)	12.5 (10.4-14.6)	45.9 (45.5-46.4)	36.2 (33.4-39.0)

**Sex**							

Male	40,325	2153	23.4 (22.7-24.1)	13.8 (10.1-17.5)	47.8 (47.0-48.6)	35.3 (31.0-39.6)
Female	64,408	3527	21.6 (21.1-22.1)	11.7 (9.4-14.0)	44.5 (43.8-45.2)	37.2 (33.6-40.8)

**Age, y**							

50–59	41,155	2459	18.1 (17.5-18.7)	9.8 (7.5-12.1)	37.2 (36.4-38.0)	25.3 (21.9-28.7)
60–69	29,150	1731	24.7 (23.8-25.6)	13.5 (10.5-16.5)	49.7 (48.7-50.7)	37.7 (33.0-42.4)
70–79	23,449	1106	27.4 (26.4-28.4)	16.0 (9.5-22.5)	55.5 (54.4-56.6)	46.9 (38.9-54.9)
≥80	10,979	384	22.4 (21.1-23.7)	13.1 (5.8-20.4)	49.4 (47.7-51.1)	50.5 (40.1-60.9)

**Education**							

<High school	14,422	2242	16.8 (15.6-18.0)	6.4 (4.3-8.5)	34.7 (33.2-36.2)	30.2 (26.0-34.4)
High school graduate	35,081	1645	21.1 (20.4-21.8)	18.2 (13.2-23.2)	42.0 (41.1-42.9)	36.5 (31.9-41.1)
>High school	54,951	1774	24.4 (23.8-25.0)	17.3 (13.4-21.2)	51.0 (50.3-51.7)	45.1 (40.3-49.9)

**Annual household income, $**							

<20,000	2,176	2650	19.2 (18.2-20.2)	6.5 (4.7-8.3)	36.7 (35.5-37.9)	32.0 (28.1-35.9)
20,000–34,999	23,114	1097	21.1 (20.3-21.9)	17.7 (12.3-23.1)	42.1 (41.0-43.2)	39.6 (33.8-45.4)
35,000–74,999	23,916	849	23.5 (22.6-24.4)	22.8 (15.8-29.8)	50.0 (48.9-51.1)	37.2 (29.6-44.8)
≥75,000	14,237	272	26.7 (25.1-28.3)	21.4 (13.3-29.5)	57.1 (55.3-58.9)	56.5 (47.4-65.6)

**Insurance**							

Yes	96,838	4943	23.0 (22.5-23.5)	14.0 (11.7-16.3)	47.2 (46.7-47.7)	38.5 (35.5-41.5)
No	7,727	730	13.8 (11.9-15.7)	—d	27.9 (25.5-30.3)	20.3 (13.5-27.1)

**Usual source of health care**							

Yes	94,595	4792	23.5 (23.0-24.0)	14.3 (12.0-16.6)	47.9 (47.3-48.5)	40.4 (37.5-43.3)
No	9,915	880	10.5 (9.4-11.6)	6.5 (2.3-10.7)	26.8 (24.8-28.8)	21.5 (15.3-27.7)

a Age-adjusted to the 2000 U.S. census.

b FOBT indicates fecal occult blood test.

c CI indicates confidence interval.

d Unstable estimate (relative SE >30%).

**Table 3:** Adjusted^a^ Odds Ratios (ORs) of Receiving Colorectal Cancer Tests, by Hispanic Ethnicity, Education, and Health Care Access, Behavioral Risk Factor Surveillance System, 2002

**Characteristic**	**FOBT^b^ Within Past YearAdjusted OR (95% CI^c^)**	**Lower Endoscopy Within 10 YearsAdjusted OR (95% CI^c^)**

**Ethnicity**		

Non-Hispanic	1.00 (ref^d^)	1.00 (ref)
Hispanic	0.66 (0.56-0.81)	0.87 (0.77-0.99)

**Education**		

>High school	1.00 (ref)	1.00 (ref)
High school graduate	0.89 (0.83-0.94)	0.76 (0.73-0.80)
<High school	0.65 (0.60-0.72)	0.63 (0.58-0.68)

**Insurance**		

Yes	1.00 (ref)	1.00 (ref)
No	0.63 (0.56-0.72)	0.63 (0.56-0.70)

**Usual source of health care**		

Yes	1.00 (ref)	1.00 (ref)
No	0.41 (0.36-0.47)	0.43 (0.39-0.47)

a Logistic regression model included age, sex, race, Hispanic ethnicity, education, income, insurance, and having a usual source of health care.

b FOBT indicates fecal occult blood test.

c CI indicates confidence interval.

d Ref indicates referent group.

**Table 4:** Percentage^a^ of Hispanic Respondents Aged 50 Years and Older Who Reported Receiving Fecal Occult Blood Test (FOBT), Lower Endoscopy, or Both Within Recommended Intervals, by Area and Test Type, Behavioral Risk Factor Surveillance System, 2002

**Area**	**Hispanic Ethnicity, % of U.S. Population (2000 Census)**	**No. of Hispanic Respond-ents**	**FOBT Within 1 Year, % (95% CI^b^)**	**Lower Endoscopy Within 10 Years, % (95% CI^b^)**	**FOBT Within 1 Year or Lower Endoscopy Within 10 Years, % (95% CI^b^)**

**Northeast**					

Massachusetts	6.8	148	24.1 (16.3-31.9)	47.1 (38.5-55.7)	61.5 (52.8-70.2)
New York	15.1	103	16.7 (9.0-24.4)	37.3 (27.2-47.4)	49.5 (37.7-61.3)
New Jersey	13.3	110	13.6 (7.3-19.9)	41.8 (29.0-54.6)	51.4 (40.2-62.6)

**South**					

Florida	16.8	196	17.2 (10.8-23.6)	41.5 (34.1-48.9)	46.7 (39.1-54.3)
Texas	32.0	334	8.5 (5.4-11.6)	31.2 (25.1-37.3)	38.2 (32.0-44.4)

**West**					

Arizona	25.3	129	21.2 (12.6-29.8)	34.8 (26.3-43.3)	47.1 (37.8-56.4)
California	32.4	180	11.2 (5.3-17.1)	37.6 (29.6-45.6)	40.3 (32.1-48.5)
Colorado	17.1	135	15.8 (10.1-21.5)	28.9 (21.4-36.4)	35.7 (27.7-43.7)
New Mexico	42.1	600	13.6 (10.4-16.8)	36.4 (31.7-41.1)	43.6 (38.8-48.4)

**Territory**					

Puerto Rico	98.8	1873	—c	30.0 (27.4-32.6)	30.3 (27.7-32.9)

a Age-adjusted to 2000 U.S. census.

b CI indicates confidence interval.

c Unstable estimate (relative SE >30%)
